# Antidiarrheal Activity of 19-Deoxyicetexone Isolated from *Salvia ballotiflora* Benth in Mice and Rats

**DOI:** 10.3390/molecules18088895

**Published:** 2013-07-26

**Authors:** Salud Pérez-Gutiérrez, Daniel Zavala-Mendoza, Abigail Hernández-Munive, Ángel Mendoza-Martínez, Cuauhtemoc Pérez-González, Ernesto Sánchez-Mendoza

**Affiliations:** 1 Departamento de Sistemas Biológicos, Universidad Autónoma Metropolitana. Unidad Xochimilco, Calzada del Hueso 1100, Col. Villa Quietud, Coyoacán 04960, Mexico; 2 Doctorado en Biología Experimental. D.C.B.S. Universidad Autónoma Metropolitana. Unidad Iztapalapa, Av. San Rafael Atlixco No. 186 Col. Vicentina, Iztapalapa 09340, Mexico; 3 Centro de Química, ICUAP, Benemérita Universidad Autónoma de Puebla, Ciudad Universitaria Puebla 72592, Mexico

**Keywords:** *Salvia ballotiflora*, antidiarrheal activity, 19-deoxyicetexone

## Abstract

The antidiarrheal properties of 19-deoxyicetexone, a diterpenoid isolated from *Salvia ballotiflora* were evaluated on castor oil-, arachidonic acid (AA)- and prostaglandin (PGE_2_)-induced diarrhea in rodent models. The structure of 19-deoxyicetexone was determined by X-ray crystallography, mass spectrometry (EI-MS), as well as ultraviolet (UV-Vis), infrared (FT-IR) and nuclear magnetic resonance (NMR) spectroscopies. This compound significantly and dose-dependently reduced frequency of stooling in castor oil-induced diarrhea, and at dose of 25 mg/kg it also inhibited diarrhea induced with AA, while it had no effect on PGE_2_-induced diarrhea. This compound at doses of 25 mg/kg also diminished castor oil-induced enteropooling and intestinal motility, and inhibited the contraction of the rats’ ileum induced by carbachol chloride at a concentration of 100 µg/mL. 19-Deoxyicetexone did not present acute toxicity at doses of 625 mg/kg. Its antidiarrheal activity may be due to increased reabsorption of NaCl and water and inhibition of the release of prostaglandins, gastrointestinal motility and fluid accumulation in the intestinal tracts of rats. These findings suggest that 19-deoxyicetexone may be used in the treatment of diarrhea, although more studies must be carried out to confirm this.

## 1. Introduction

Diarrheal disease is one of the main causes of the high mortality rate in developing countries, particularly among children under the age of five [1]. Diarrhea is most common in crowded living conditions coupled with poor hygiene and malnutrition [2]. Thus, it is important to identify and evaluate natural drugs that can be used as alternatives to commonly used antidiarrheal drugs, which are often accompanied by adverse effects such as addiction and constipation [3]. Consequently, much research is now being devoted to the discovery of new antidiarrheal compounds isolated from natural products. In some cases, these compounds may be more specific and less toxic than those obtained by synthesis. Numerous plants from all continents have traditionally been used in the treatment of diarrheal disease [4].

According to World Health Organization (WHO) about 80% of the World’s population depends mainly on traditional medicine and the use of plant extracts are involved in these traditional treatments [5], mainly due to the economic viability, accessibility and ancestral experience [6]. In particular, Mexico has great environmental and biological diversity and is home to a wide range of medicinal plants with antidiarrheal properties have been used by traditional healers. However, the effectiveness of many of these plants has not been scientifically evaluated, and the compounds responsible for the antidiarrheal effects of these plants have not been characterized in most cases.

*Salvia ballotiflora* Benth (Acanthaceae) is commonly known in Mexican traditional medicine as *mejorana* and is a multi-branched aromatic shrub with square stems. The leaves of the plant contain serrated margins and have hairs on the top and bottom. The flowers of the plant are bluish-purple in color and grow in elongated clusters. This plant has been used by the indigenous people of Mexico to relieve postpartum symptoms [7].

In 1997, Esquivel *et al* [8] isolated three icetexane diterpenoids from the aerial parts of *Salvia ballotiflora*, and their chemical structures were elucidated using spectroscopic methods. These compounds were identified as 19-deoxyicetexone, 19-deoxyisoicetexone and 7,20-dihydroanastomosine.

The aim of the present study was to obtain and conform the identity of the diterpenoid 19-deoxyicetexone isolated from *Salvia ballotiflora* and evaluate for the first time its antidiarrheal properties in castor oil-, arachidonic acid (AA)- and prostaglandin (PGE_2_)-induced rodent diarrhea models as well as its effects on intestinal transit and castor oil-induced enteropooling.

## 2. Results and Discussion

### 2.1. Structural Analyses

**Figure 1:**
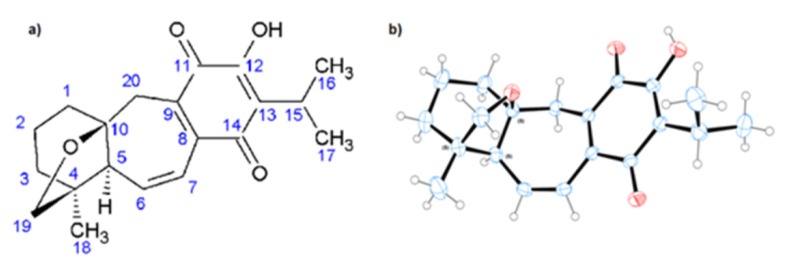
(**a**) Structure of 19-deoxyicetexone. (**b**) X-ray ORTEP diagram of 19-deoxyicetexone.

The chemical structure of 19-deoxyicetexone was unequivocally determined using X-ray crystallography (Figure 1a) by making use of the anomalous scattering of Cu K_α_ radiation with the Flack parameter being refined to 0.09 (19). The absolute configuration of compound revealed three chiral centers: C8=S, C10=S, and C11=S (Figure 1b).

The crystal structure of 19-deoxyicetexone was deposited at the Cambridge Crystallographic Data Centre (deposition number: CCDC 917975). *Crystal data*: C_20_H_24_O_4_, orange needles, T = 293 (2) K, Cu *Κα* λ = 1.54184 Å, crystal system orthorhombic, space group *P*2_1_2_1_2_1_, unit cell dimensions: *a* = 7.7141 (1) Å, *b* = 10.5176 (2) Å, and *c* = 20.7343 (3) Å, α = β = γ = 90°, *V* = 1682.25 (5) Å^3^. Z = 4, *Dx* = 1.297 mg m^−3^, µ=0.72 mm^−1^, and F(000) = 704. Collected reflections: 12117; independent reflections: 3443 in a *θ_max_* = 77.1° and *θ_min_* = 4.3° (−6 ≤ *h* ≥ 9, −13 ≤ *k* ≥ 13, −26 ≤ *l* ≥ 25). Final *R* indices (all data), *R*1 = 0.045 and *wR*2 = 0.121.

19-Deoxyicetexone was previously reported by Esquivel *et al.* [8]. Our ^13^C-NMR, EI-MS and infrared data are consistent with the previously reported data. However, there were some discrepant results: the melting point in the present study was 203–204 °C, the optical rotation [α]_D_ = −66.81, and the UV 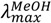 264 and 317 nm (as opposed to mp 228–230 °C, [α]^20^ = +95 and the UV 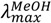 211, 290, and 318 nm as previously reported). In addition, we add to the scientific record the complete ^1^H-NMR assignment of 19-deoxyicetexone (Table 1).

**Table 1:** NMR spectroscopy data for 19-deoxyicetexone (600 MHz. CDCl_3_).

Position	δ^13^C (ppm)	δ^1^H (ppm)	Position	δ^13^C (ppm)	δ^1^H (ppm)
1	39.88 (39.8) *	1.76 (m), 1.70 (m)	12	150.75 (150.7) *	-
2	20.19 (20.2) *	1.89 (m), 1.68 (m)	13	124.48 (124.4) *	-
3	39.19 (39.1) *	1.57(m), 1.50 (tdd)	14	186.54 (186.5) *	-
4	43.90 (43.9) *	-	15	24.30 (24.3) *	3.22 (hept)
5	59.30 (59.3) *	2.31 dd	16	19.92 (19.9) *	1.24 (d)
6	141.82 (141.8) *	6.51 dd	17	19.88 (19.9) *	1.23 (d)
7	124.13 (124.1) *	6.81 dd	18	20.03 (19.5) *	1.04 (s)
8	135.63 (135.5) *	-	19	77.72 (77.6) *	3.70 (d), 3.50 (dd)
9	139.95 (138.6) *	-	20	33.23 (33.2) *	2.98 (d), 2.59 (d)
10	92.91 (92.9) *	-	OH	-	7.17 (s)
11	183.31 (183.3) *	-			

* ^13^C-NMR data were reported by Esquivel *et al.* [8].

### 2.2. Pharmacological Study of 19-Deoxyicetexone

#### 2.2.1. Acute Toxicity

Oral administration of 19-deoxyicetexone at doses of 312.5 or 625 mg/kg produced no visible signs of toxicity (for example, restlessness and seizures) in the animals. No deaths were recorded following the administration of these two doses.

#### 2.2.2. Antidiarrheal Activity

Thirty minutes after castor oil administration, diarrhea was clinically apparent in all animals of the group administered only with vehicle for the next 4 h. Table 2 shows that loperamide administration at a dose of 2.5 mg/kg markedly reduced diarrhea (81.5 ± 4.5) when compared to the untreated rats. A similar reduction in the total number of defecations over a 4 h, period was achieved with 19-deoxyicetexone at doses of 12.5 and 25 mg/kg (61.8 and 89.6%, respectively). However, at doses of 6.25 mg/kg this compound did not affect the severity and onset diarrhea.

**Table 2:** The antidiarrheal effects of 19-deoxyicetexone on mice induced castor oil-induced diarrhea.

Treatment	Doses mg/kg	Percentage of inhibition
Vehicle	0.1 mL	0.0
19-Deoxyicetexone	6.25	30.9 ± 7.2 ^ns^
	12.5	61.8 ± 5.5 *
	25	89.6 ± 1.7 **
Loperamide	2.5	81.5 ± 4.5 **

The results are the means of 10 animals ± standard error. * *p* < 0.05 and ** *p* < 0.001 with respect to the vehicle-treated group.

It is well known that castor oil produces diarrhea due to the most active metabolite ricinoleic acid which causes irritation and inflammation of the intestinal mucosa, leading to release of prostaglandins [9,10] and nitric oxide [11,12] which stimulate the intestinal motility, secretion and diminish the reabsorption of NaCl and H_2_O [13,14]. The results of the present study indicate that 19-deoxyicetexone produced a statistically significant reduction in the severity and frequency of castor oil-induced diarrhea.

For this reason the activity of 19-deoxyicetexone was tested on mice with AA- or PGE_2_-induced diarrhea. The inhibitory effect of the compound on AA-induced diarrhea at a dose of 25 mg/kg was 75.0 ± 2.3%, which was similar to that observed with loperamide at doses of 2.5 mg/kg (77.8 ± 4.5%) (Table 3). 19-Deoxyicetexone produced a significant inhibition of AA-induced diarrhea but this compound had no effect on PGE_2_-induced diarrhea, suggesting that 19-deoxyicetexone inhibited the release of prostaglandins [15] and that this diterpenoid shows affinity and selectivity for the opioid receptor [16].

**Table 3:** The antidiarrheal effects of 19-deoxyicetexone on mice induced AA- or PGE_2_-induced diarrhea.

Cathartic agent	Treatment	Doses mg/kg	Percentage of inhibition
Arachidonic acid	Vehicle	0.1 mL	0.0
	19-Deoxyicetexone	25	75.0 ± 2.3 **
	Loperamide	2.5	77.8 ± 4.5 **
Prostaglandins E_2_	Vehicle	0.1 mL	0.0
	19-Deoxyicetexone	25	22.2 ± 9.1 ^ns^
	Loperamide	2.5	69.4 ± 2.3**

The results are the means of 10 animals ± standard error. ** *p* < 0.001 with respect to the vehicle-treated group.

#### 2.2.3. Ileum Contractions

**Figure 2:**
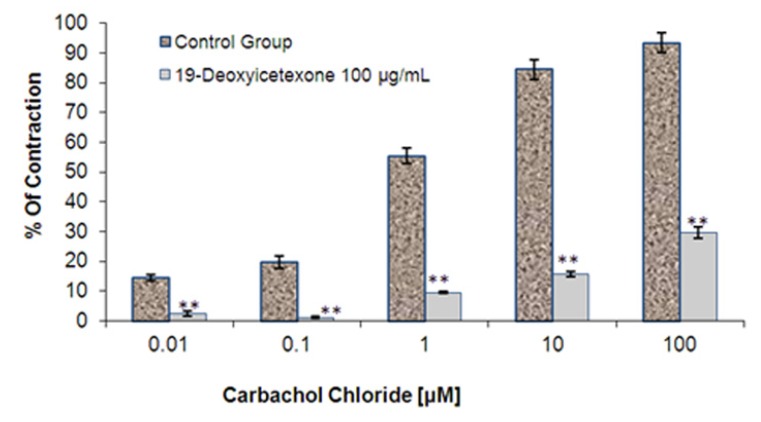
Effect of 19-deoxyicetexone (100 μg/mL) on the contraction of ileum stimulated with carbachol chloride. The results are the mean of six determinations ± standard error. ** *p* < 0.001 with respect to the carbachol chloride-treated.

The contractile response obtained by stimulating a strip of the longitudinal layer of the Wistar rat ileum with carbachol chloride, which was applied at concentrations of 0.01, 0.1, 1.0, 10 and 100 µM was evaluated (Figure 2). The peak amplitude of the successive carbachol-induced contractions declined when 100 µg/mL of 19-deoxyicetexone was added. This compound reduced the contractile force by almost 68%, suggesting that 19-deoxyicetexone is a competitive blocker of carbachol chloride and that this effect may be due to the interaction of 19-deoxyicetexone with the carbachol chloride muscarinic receptor (μ3) [17,18].

#### 2.2.4. Small Intestinal Transit

**Figure 3:**
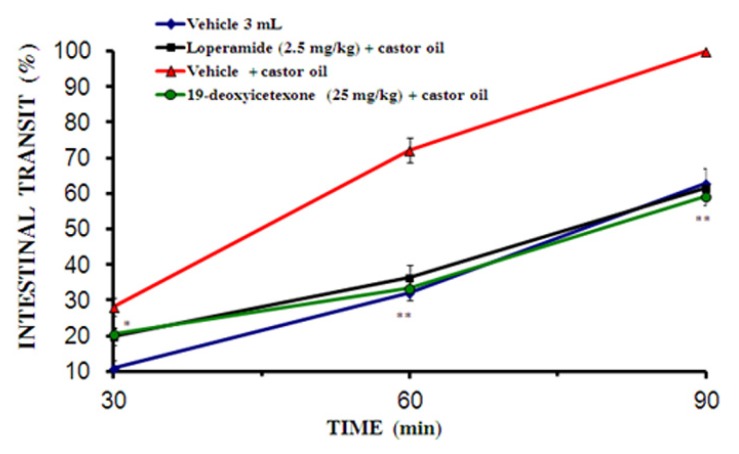
Effects of 19-deoxyicetexone (25 mg/kg) on intestinal transit in rats. The results are the means of five determinations ± standard error. * *p* < 0.05 with respect to the castor oil-treated group.

The charcoal meal moved farther during castor oil-induced intestinal transit compared to normal intestinal transit. In the presence of castor oil, the percent of intestinal transit was increased (100%) after 90 min (Figure 3), but was reduced in the presence of loperamide at dose of 2.5 mg/kg (61%) and 19-deoxyicetexone at dose of 25 mg/kg (60%). This result might be due to its anticholinergic effects [18].

#### 2.2.5. Castor Oil-Induced Enteropooling

Administration of castor oil resulted in the accumulation of water and electrolytes in the intestinal loop. 19-Deoxyicetexone (25 mg/kg) administration significantly inhibited castor oil-induced intestinal fluid accumulation in rats (53.3% of weight) to a value that was similar to that obtained with loperamide (60%) (Table 4).

**Table 4:** Effect of 19-deoxyicetexone on castor oil induced intestinal fluid accumulation.

Treatment	Dose (mg/kg)	Wt of intestinal content (g)	% Inhibition
Control (vehicle)	2 mL	0.5 ± 0.3	
Castor oil	2 mL	3.0 ± 0.06	
19-Deoxyicetexone	25	1.4 ± 0.2 *	53.3
Loperamide	2.5	1.2 ± 0.3 *	60.0

The results are the mean of 10 animals ± standard error; ** *p* < 0.005 with respect to the castor oil-treated group.

Castor oil produced accumulation of water and electrolytes in the intestinal loop. 19-Deoxyicetexone inhibited castor oil-induced intestinal fluid accumulation in rats. The mechanism involved has been associated with the dual effects of gastrointestinal motility as well as water and electrolyte transport across the intestinal mucosa [19]. Thus, it is possible to suggest that 19-deoxyicetexone reduced diarrhea by increasing reabsorption of electrolytes and water or by inhibiting induced intestinal accumulation of fluid just like the standard drugs such as loperamide.

The results of this study indicate that 19-deoxyicetexone has antidiarrheal activity, and this might be due to its effect on reduction of water and electrolytes, inhibition release of PGE_2_ reduction number of gastrointestinal motility and fluid accumulation in the intestinal tract of rats, the acute toxicity is very low. No damage was observed on intestine, liver and kidney. Thus, this compound could potentially be used in the treatment of diarrhea.

## 3. Experimental

### 3.1. Plant Material

*Salvia ballotiflora* was collected in Las Comadres Municipality of Guadalcazar, San Luis Potosi State, México in July of 2010. Vegetal identification was performed by taxonomist José García Pérez. A voucher specimen (SLPM 43013) was deposited at the herbarium Isidro Palacios Herbarium of the Universidad Autónoma de San Luis Potosí.

### 3.2. Extraction and Isolation of 19-Deoxyicetexone

The dried and ground plant (854 g) was extracted with CHCl_3_ (5 L) at its boiling point for 4 h. The solvent was removed under vacuum to yield 20.7 g of a gummy residue (2.42%). Ten grams of this extract were fractionated in an open chromatographic column packed with silica gel 60. Mixtures of hexane-ethyl acetate with increasing polarities were used for fractionation. The final solution used was 100% ethyl acetate. The fractionation process was monitored using thin layer chromatography. The isolated compound was obtained from fraction 8 (176 mg). This compound was named 19-deoxyicetexone and is an orange crystalline solid with a melting point of 203–204 °C and an optical rotation of [α]^20^ = −66.81 (c 0.2,CHCl_3_).

### 3.3. Structural Analysis of 19-Deoxyicetexone

Structural identification was performed using X-ray crystallography, and UV-Vis, FT-IR, and NMR spectroscopies. The molecular weight was obtained by EI-MS. The crystallographic data were collected on an Oxford Gemini-Atlas diffractometer with Cu Κ_α_ radiation (λ = 1.54184 Å) at 298 (2) K. The mass spectrum was recorded using the Electronic Ionization (EI) method in the positive mode by direct injection on a Jeol-MStation mass spectrophotometer. The UV spectrum was obtained in a CHCl_3_ solution on a Shimadzu spectrophotometer double beam model UV-1800. The IR spectrum was recorded with an ATR accessory using a Perkin-Elmer Paragon 1000 FT-IR spectrophotometer. NMR spectra were recorded in a CDCl_3_ solution at 299 K on an Agilent DD2 600 spectrometer. The ^1^H and ^13^C NMR chemical shifts were reported relative to TMS and CDCl_3_, respectively.

### 3.4. Antidiarrheal Activity

#### 3.4.1. Animals

Male CD1 mice (20–25 g) and male Wistar rats (200–250 g) were obtained from the Universidad Autónoma Metropolitana animal facility, and housed in isolated cages under standardized conditions (dark/light 12/12) at 30 °C and 50%–55% humidity. They were supplied with Pet Food 5001 (LabDiet^®^, Richmond, IN, USA) and water *ad libitum*. The animals were submitted to a fasting period of 18–24 h with free access to water prior the study. All the experiments were performed according to the international guidelines for the care of laboratory animals; they were sacrificed by cervical dislocation according to the international guidelines for the care of laboratory animals [20].

#### 3.4.2. Evaluation of Antidiarrheal Activity

Groups of 10 mice were orally administered either the compound (6.25, 12.5 or 25 mg/kg), loperamide (2.5 mg/kg), or vehicle (0.1 mL) 30 min prior to the oral administration of castor oil (4 mL/kg) [21,22]. Following castor oil treatment, the animals were placed separately in acrylic cages with filter paper at the bottom, which was changed every hour. The severity of diarrhea was assessed every hour for 4 h. The total amount of watery feces excreted was scored and compared with the score obtained in the control group. The total diarrheic feces score from the control group was considered to be 100%. The results are expressed as the percentage of inhibition.

#### 3.4.3. Evaluation of Antidiarrheal Activity in AA- and PGE2-Induced Diarrhea

After evaluating the activity using the models described above, the effects of 19-deoxyicetexone at a dose of 25 mg/kg were evaluated on AA- (3 mg/kg) or PGE_2_- (1 mg/kg) induced diarrhea in accordance with the methods described by Melo *et al.* [23].

#### 3.4.4. Evaluation of the Effects of 19-Deoxyicetexone on Ileum Contractions

Male Wistar rats (300 g) were sacrificed by cervical dislocation according to the international guidelines for the care of laboratory animals [20]. The abdomens were opened and segments of the ileum (10 cm proximal to the caecum) were flushed twice with an aerated physiological salt solution (PSS) to remove their contents. The ileum segments were cut into smaller segments (1 cm long approximately), which were placed in a 2 mL organ bath containing PSS with the following composition (mM): NaCl (118), NaHCO_3_ (25), KCl (4.7), KH_2_PO_4_ (1.2), MgSO_4_ (1.2), CaCl_2_ (2.5) and D-glucose (11). The organ bath was maintained at 36 °C while being aerated by bubbling with a mixture of 95% O_2_ and 5% CO_2_ (pH 7.4). The ileum contractions were recorded isometrically with a Grass FT03 force displacement transducer connected to a Grass TBRS2 polygraph. The tissues were allowed to equilibrate for 60 min, during which time the PSS was changed every 20 min and maintained under an optimal tension of 1 g prior to the initiation of experiment. After equilibration, the ileum rings were bathed in a depolarization solution (23 mM KCl) that was prepared by equimolecular substitution of NaCl for KCl to expose the tissues to a single sub-maximal concentration of KCl (23 mM) and elicit contractile activity. Control contractile responses were considered to be two successive similar responses.

A group of tissues (n = 6) were incubated 10 min with solvent (ethanol-dimethyl sulfoxide 1:1, 20 µL) corresponding to 1% in the bath. Each group of tissues was stimulated with a different concentration of carbachol chloride (0.01, 0.1, 1.0, 10 and 100 µM), the amplitude was measured. After that, each tissue was incubated with 19-deoxyicetexone (100 µg/mL) and then was added above concentrations of carbachol chloride and the amplitude was recorded according to the model described by Estrada *et al.* [24]. The contractile response for each tissue treated with carbachol chloride was considered to be a response of 100% and was compared with the contractile response of the tissues that had been pretreated with 19-deoxyicetexone.

#### 3.4.5. Small Intestinal Transit

Small intestinal transit was evaluated according to the method described by Visher *et al.* [25]. A 2.0% suspension of graphite in 1.5% agar was administered orally to groups of 15 rats (1.5 mL/animal). The inhibitory action of the extracts on stimulated intestinal transit was tested using the following procedure: Castor oil (4 mL/kg) was administered along with a graphite-agar suspension 60 min after the vehicle or the extract had been administered. At 30, 60 and 90 min after the administration of the graphite-agar suspension plus castor oil, the rats were killed in groups of five and the gastrointestinal tracts were removed and opened. Distance traveled by the marker was measured and expressed as a percentage of the total length of the intestine from pylorus to caecum.

#### 3.4.6. Castor Oil-Induced Enteropooling

Intraluminal fluid accumulation was evaluated according to the method described by Robert *et al.* [26]. Overnight fasted rats were divided in groups of six animals each, they were administered orally. Group 1 received vehicle (1 mL/rat), group 2 received loperamide (2.5 mg/kg), and group 3 received 19-deoxyicetexone (25 mg/kg) 1 h prior to the oral administration of castor oil (2 mL/rat). Two hours later, the rats were sacrificed, the small intestines were removed after tying the ends, the intestinal contents were collected by milking into a graduated tube and their weights were measured.

#### 3.4.7. Acute Toxicity

19-Deoxyicetexone was orally administered as a single dose to groups of mice (n = 5) at doses of 625 and 312 mg/kg. The range of doses used in mice was based upon the OECD method [27]. After administration, the animals were observed under open-field conditions for a 72 h period. The number of animal deaths and all signs of clinical toxicity were recorded. Intestine, liver, and kidney were grossly examined.

#### 3.4.8. Statistical Analyses

The results are expressed as the means ± s.e.m. The mean values of intestinal transit were evaluated using Student’s *t*-test, and the antidiarrheal activity, effect on inhibition of ileum contractile activity and castor oil-induced enteropooling were analyzed using ANOVA followed by Tukey’s *post-hoc* test. The tests were used to compare the mean values of the activity of each group with the control group. The statistical significance was set at *p* < 0.05.

## 4. Conclusions

The results of the present study suggest that 19-deoxyicetexone has antidiarrheal activity, which may be due to increased reabsorption of NaCl and water and inhibition of the release of prostaglandins, as well as inhibition of gastrointestinal motility and fluid accumulation in the intestinal tracts of rats and the mechanisms of this compound might involve inhibition of enteric neurotransmission, however, further studies must be carried out to verify this. Thus, this compound could potentially be used for the treatment of diarrhea.
